# Anti-Inflammatory Properties of Cannabidiol and Beta-Caryophyllene Alone or Combined in an In Vitro Inflammation Model

**DOI:** 10.3390/ph17040467

**Published:** 2024-04-07

**Authors:** Costanza Mazzantini, Zahraa El Bourji, Carmen Parisio, Pier Luigi Davolio, Arianna Cocchi, Domenico E. Pellegrini-Giampietro, Elisa Landucci

**Affiliations:** 1 Department of Health Sciences, Section of Clinical Pharmacology and Oncology, University of Florence, Viale Pieraccini 6, 50139 Florence, Italy; costanza.mazzantini@unifi.it (C.M.); zahraa.elbourji@unifi.it (Z.E.B.); domenico.pellegrini@unifi.it (D.E.P.-G.); 2 Farmacia del Madonnone, Via Aretina 9R, 50135 Florence, Italy; carmen.parisio@unifi.it (C.P.); farmaciadavolio@libero.it (P.L.D.); 3 Tuscopharm srl, Viale Giacomo Leopardi 45, 57121 Livorno, Italy; rd02@pediatricaspecialist.it

**Keywords:** human keratinocytes (HaCaT cells), lipopolysaccharide (LPS), cyclooxygenase-2 (COX-2), interleukin-1β (IL-1β), phospho-NF-κB p65 (p-p65), interleukin-6 (IL-6), tumor necrosis factor (TNFα)

## Abstract

Cannabis contains over 500 different compounds, including cannabinoids, terpenoids, and flavonoids. Cannabidiol (CBD) is a non-psychoactive constituent, whereas beta-caryophyllene (BCP) is one of most the well-known terpenoids of *Cannabis sativa*. In recent years, there has been an emerging idea that the beneficial activities of these compounds are greater when they are combined. The aim of this study was to evaluate the anti-inflammatory effect of CBD and BCP using the in vitro model of lipopolysaccharide (LPS)-stimulated human keratinocytes (HaCaT) cells. The vitality of the cells was quantified using LDH and MTT assays. The levels of the following pro-inflammatory proteins and genes were quantified: IL-1β, COX-2, and phospho-NF-κB p65 (p-p65) through Western blotting (WB) and interleukin-1β (IL-1β), interleukin-6 (IL-6), and tumor necrosis factor-α (TNFα) through quantitative real-time polymerase chain reaction (RT-qPCR). When present in the incubation medium, CBD and BCP reduced the increased levels of pro-inflammatory proteins (IL-1β, COX-2, and p-NF-kB) induced by LPS. The anti-inflammatory effects of CBD were blocked by a PPARγ antagonist, whereas a CB2 antagonist was able to revert the effects of BCP. Selected concentrations of CBD and BCP were able to revert the increases in the expression of pro-inflammatory genes (IL-1β, IL-6, and TNFα), and these effects were significant when the drugs were used in combination. Our results suggest that CBD and BCP work in concert to produce a major anti-inflammatory effect with good safety profiles.

## 1. Introduction

In the last few years, with the continuously increasing use of botanical components in dermatology, many studies have found different natural substances both effective and safe [1]. Recently, research has been focused on the therapeutic applications of *Cannabis sativa* (*C. sativa*) constituents for the modulation of the inflammatory process, related to the activation of the endocannabinoid system (ECS) [2,3]. The use of cannabis components in healthcare and skincare formulation is growing in different countries [4]. Cannabis contains more than 500 distinct compounds, which include cannabinoids, terpenoids, and flavonoids [5]. The major non-psychotropic cannabinoid is cannabidiol (CBD) [6], which has anti-inflammatory activity against various types of inflammation diseases, including airway inflammation, arthritis, intestinal inflammation, and skin inflammation [7]. Another group of important bioactive compounds found in cannabis are the terpenes, of which beta-caryophyllene (BCP) is the most abundant; it is a sesquiterpene that is also found in black peppers and cloves [8]. In the essential oil of black peppers, BCP is the main component, totaling 30% [9], compared to 23.8% in cannabis essential oil [8]. In addition, recent studies have demonstrated that BCP has anti-cancer [10], antioxidant [11], and anti-inflammatory properties [12]. The essential role of ECS dysregulation in human skin, such as in homeostasis and barrier function, atopic dermatitis, acne, and hyperpigmentation, suggests the topical use of cannabis components in the treatment of skin pathology [13].

Skin, as the outermost immune organ of the human body, serves as a protective barrier against environmental insults, which can lead to the generation of reactive oxygen species (ROS) [14,15] that cause skin damage and diseases, including aging, tumors, and inflammation [16]. Keratinocytes are the main cells in the epidermis and play multiple essential roles for skin function and repair. These cells maintain homeostasis, activate repair mechanisms after injuries, and have fundamental immune functions during inflammatory processes [17]. Indeed, keratinocytes are particularly sensitive to environmental stressors [18], and they express a broad range of pattern-recognition receptors (PRRs), including toll-like receptors (TLRs), C-type lectin receptors, nucleotide-binding oligomerization domain-like receptors, and retinoic acid-inducible gene I (RIG-I)-like receptors [19].

The most representative cells of the human epidermis are keratinocytes, and the most frequent pathologies of the skin are indicated by inflammation [20]. Lipopolysaccharides (LPS) are important components of Gram-negative bacteria [21], and they are one of the most common stimulators of inflammation [22]. LPS can induce the activation of the nuclear-factor kappa light-chain enhancer of activated B cells (NF-kB), the signaling pathway that is responsible for the release of pro-inflammatory cytokines [17], including tumor necrosis factor-α (TNF-α), interleukin-1β (IL-1β), and interleukin-6 (IL-6) [23]. It has also been demonstrated that cyclooxygenase-2 (COX-2) is inflammatory, because this enzyme induces pro-inflammatory responses to LPS [24].

TNF-α, IL-1β, and IL-6 are also targets of the therapeutic effects of CBD and BCP [25]. With the growing emphasis on the use of natural actives in different human diseases, CBD has become a new ingredient for topical application due to its function in alleviating skin inflammation [26]. Also, BCP has demonstrated effective anti-inflammatory activity in skin diseases such as atopic dermatitis (AD) [27]. BCP and CBD are natural compounds that have valid therapeutic properties with good safety profiles and minimal side effects [28]. Based on all these data, this study aimed to evaluate the anti-inflammatory effects of CBD and BCP and the possible synergism of their combination using the in vitro model of LPS-stimulated HaCaT cells, which express a complete and functional ECS system as present in the skin [29].

## 2. Results

### 2.1. Effects of Cannabidiol and Beta-Caryophyllene on Cell Viability in Human Keratinocyte (HaCaT) Cells

**Figure 1:**
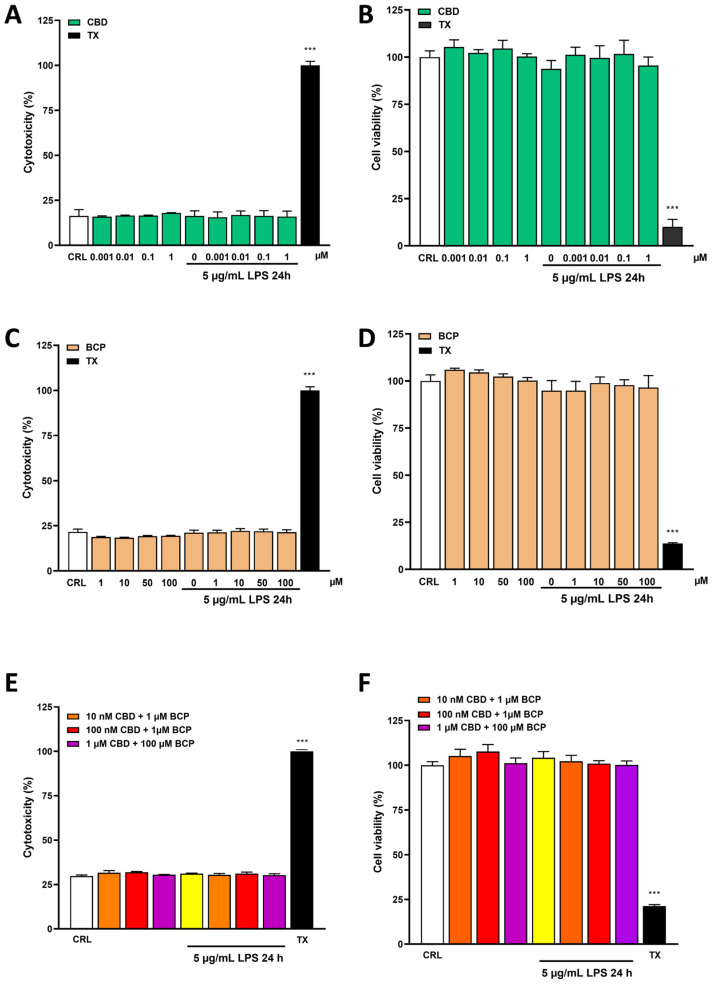
The evaluation of LDH and MTT assays in the HaCaT cells after 24 h of incubation with CBD (0.001–1 µM) or BCP (1–100 µM) alone or in combination, in the absence or presence of LPS 5 µg/mL. Data are expressed as the percentage of the maximum degree of cell death induced by Triton-X 0.01% (**A**,**C**,**E**) and as percentage of the maximum cell viability (medium) (**B**,**D**,**F**). *** *p* < 0.001 vs. CRL (one-way ANOVA plus Dunnett’s). Data are presented as the mean ± SEM of the mean of at least three experiments performed in triplicate.

HaCaT cells were exposed for 24 h to CBD (0.001–1 μM) and/or BCP (1–100 μM) alone or in combination, and sister cultures were exposed to 5 µg/mL of LPS for 24 h in the presence or absence of CBD or BCP. The cytotoxicities of CBD and BCP alone and in combination with LPS were assessed with LDH assays, and Triton-X 0.01% was considered the positive control. Figure 1A,C shows that 24 h of incubation of HaCaT cells with CBD or BCP alone or in combination (Figure 1E) without/with LPS do not induce significant differences in LDH dosages compared to the control. Even in the viability assays carried out using MTT assays, no significant differences were highlighted between the cells treated with CBD and BCP alone or in combination without/with LPS and the control, as shown in Figure 1B,D,F. From our data, no significant toxicity of any of the substances towards keratinocyte cells was highlighted for the concentrations used.

### 2.2. The Anti-Inflammatory Effects of Cannabidiol and Beta-Caryophyllene on Lipopolysaccharide (LPS)-Stimulated Human Keratinocyte (HaCaT) Cells

**Figure 2:**
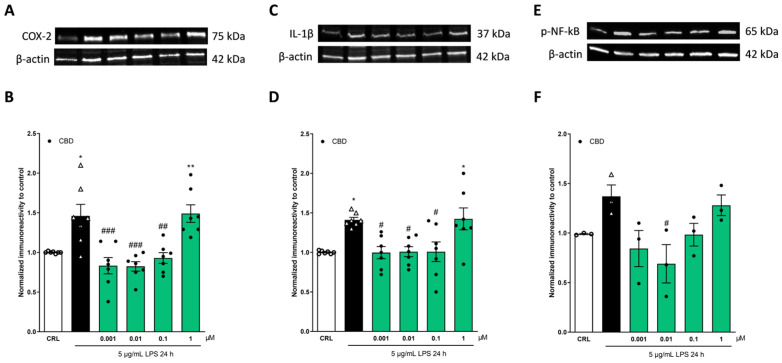
The anti-inflammatory effects of CBD on LPS-stimulated HaCaT cells. The cells were exposed to 5 µg/mL of LPS for 24 h alone or in combination with CBD, then processed for WB. Illustrative blots using antibodies directed against COX-2 (**A**), IL-1β (**C**), p-NF-kB (**E**), or β-actin. The quantitative analysis of immunoreactive bands (**B**,**D**,**F**) showed that CBD (0.001–0.1 µM) is able to reduce the increased levels of pro-inflammatory proteins induced by LPS. * *p* < 0.05 and ** *p* < 0.01 vs. CRL, # *p* < 0.05, ## *p* < 0.01, ### *p* < 0.001 vs. LPS (ANOVA + Tukey’s w-test). Δ represented LPS alone. ○ represented CRL alone.

**Figure 3:**
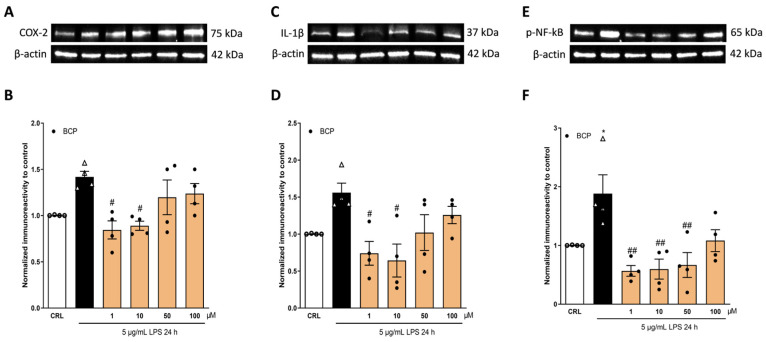
The anti-inflammatory effects of BCP on LPS-stimulated HaCaT cells. The cells were exposed to 5 µg/mL of LPS for 24 h alone or in combination with BCP and then processed using WB. Illustrative blots were created using antibodies directed against COX-2 (**A**), IL-1β (**C**), p-NF-kB (**E**), or β-actin. The quantitative analysis of immunoreactive bands (**B**,**D**,**F**) showed that the increases in the levels of pro-inflammatory proteins induced by LPS were reverted by BCP; in particular, 1 and 10 µM BCP induced a significant reduction in COX-2 (**B**), IL-1β (**D**), and p-NF-kB (**F**) levels, whereas 50 µM BCP induced a significant reduction in p-NF-kB (**F**) levels only. * *p* < 0.05 vs. CRL, # *p* < 0.05, and ## *p* < 0.01 vs. LPS (ANOVA + Tukey’s w-test). Δ represented LPS alone. ○ represented CRL alone.

In order to investigate whether CBD and BCP have anti-inflammatory effects, for 24 h, HaCaT cells were exposed to 5 µg/mL of LPS alone or in combination with different concentrations of CBD (0.001–1 µM) or BCP (1–100 µM); the total proteins were extracted, and some pro-inflammatory proteins, such as IL-1β, COX-2, and p-NFkB, were analyzed using the Western blot (WB) technique. As shown in Figure 2 and Figure 3, we observed that treatment with 5 µg/mL of LPS for 24 h induced a significant increase in the expression levels of COX-2 (Figure 2A,B and Figure 3A,B), IL-1β (Figure 2C,D and Figure 3C,D), and p-NF-kB (Figure 2E,F and Figure 3E,F) compared to the control group. Both CBD and BCP were able to reverse this effect. CBD, at the concentrations of 0.001, 0.01, and 0.1 µM, induced a significant reduction in the expression levels of COX-2 (Figure 2A,B) and Il-1β (Figure 2C,D), and 0.01 µM of CBD induced a significant reduction in p-NF-kB (Figure 2E,F). CBD showed a dose-dependent anti-inflammatory effect on HaCaT cells exposed to 5 µg/mL of LPS for 24 h. Indeed, at the concentration of 1 µM, CBD does not reduce the pro-inflammatory effect of LPS, as shown in Figure 2.

In addition, BCP induced a significant reduction in the increase in the expression levels of the inflammatory proteins induced by LPS (Figure 3). At the concentrations of 1 and 10 μM, BCP induced a significant reduction in COX-2 (Figure 3A,B), IL-1β (Figure 3C,D), and p-NF-kB levels compared to LPS (Figure 3E,F); BCP, at a 50 µM concentration, induced a significant reduction in only p-NF-kB expression levels (Figure 3).

### 2.3. The Anti-Inflammatory Effects of Cannabidiol and Beta-Caryophyllene on Peroxisome Proliferator-Activated Receptors (PPARγ) and Cannabinoid Type 2 (CB2) Receptors

**Figure 4:**
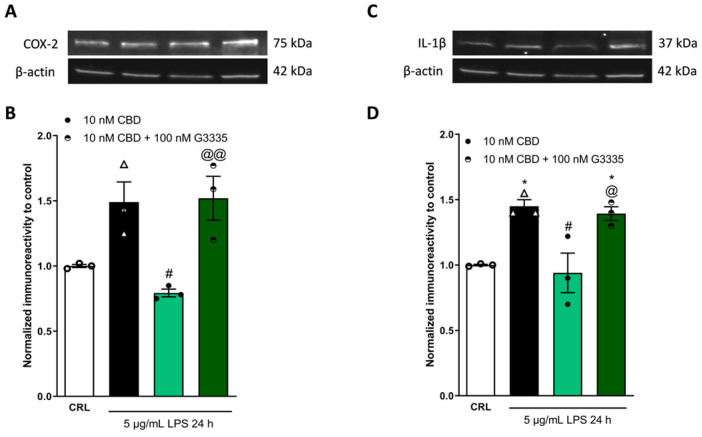
The anti-inflammatory property of CBD depends on the PPARγ receptor. Illustrative blots were created using antibodies directed against COX-2 (**A**), IL-1β (**C**), or β-actin. The quantitative analysis of immunoreactive bands (**B**,**D**) showed that the increased levels of pro-inflammatory proteins COX-2 (**B**) and IL-1β (**D**), induced on HaCaT cells by 5 µg/mL of LPS for 24 h, are reduced by 10 nM of CBD. The PPARγ antagonist G3335 blocks the anti-inflammatory effect of CBD (**B**,**D**). * *p* < 0.05 vs. CRL, # *p* < 0.05 vs. LPS, @ *p* < 0.05 and @@ *p* < 0.01 vs. CBD. (ANOVA + Tukey’s w-test). Δ represented LPS alone. ○ represented CRL alone.

The cannabinoids from *C. sativa* are ligands for cannabinoid receptor type 1 (CB1) and CB2 receptors. The psychomodulatory effects are dependent on the CB1 receptor, whereas the activation of the CB2 receptor is a potential therapeutic strategy for the treatment of inflammatory diseases. While the Δ9-tetrahydrocannabinol targets the CB receptors, the cannabidiol has no direct action on these receptors [30]. In our previous studies, we demonstrated that CBD has a neuroprotective effect mediated by transient receptor potential vanilloid (TRPV2), serotonin receptor (5-HT1A), and peroxisome proliferator-activated receptors (PPARγ) [31,32]. Instead, the mechanism of action of BCP is largely dependent on the CB2 receptor [27,33,34]; BCP is a functional CB2 agonist [35]. In order to investigate the mechanism by which CBD and BCP have an anti-inflammatory effect, HaCaT cells were incubated with 5 µg/mL of LPS for 24 h, in the presence or absence of 10 nM CBD, alone or in combination with the PPARγ antagonist G3335 at a concentration of 100 nM [32], or in the presence of 10 µM BCP, alone or in combination with 100 nM AM630, the antagonist of the CB2 receptor [33]. As shown in Figure 4, we observed that 100 nM G3335, a PPARγ antagonist, was able to prevent the anti-inflammatory effect of CBD, inducing a significant increase in the expression levels of COX-2 (Figure 4A,B) and IL-1β (Figure 4C,D), in comparison to only 10 nM CBD.

**Figure 5:**
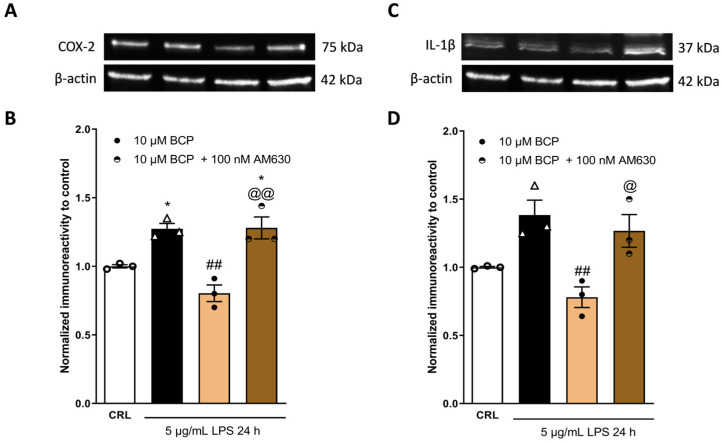
The anti-inflammatory property of BCP depends on the CB2 receptor. Illustrative blots were created using antibodies directed against COX-2 (**A**), IL-1β (**C**), or β-actin. The quantitative analysis of immunoreactive bands (**B**,**D**) showed that the increased levels of pro-inflammatory proteins COX-2 (**B**) and IL-1β (**D**), induced on HaCaT cells by 5 µg/mL of LPS for 24 h, are reduced by 10 µM BCP. The CB2 antagonist AM630 blocks the anti-inflammatory effect of BCP. * *p* < 0.05 vs. CRL, ## *p* < 0.01 vs. LPS, @ *p* < 0.05 and @@ *p* < 0.01 vs. BCP (ANOVA + Tukey’s w-test). Δ represented LPS alone. ○ represented CRL alone.

Figure 5 shows that the CB2 receptor antagonist AM630, at a concentration of 100 nM, blocked the anti-inflammatory effect of BCP, increasing the expression levels of COX-2 (Figure 5A,B) and IL-1β (Figure 5C,D).

### 2.4. Investigation of the Additive Effects

**Figure 6:**
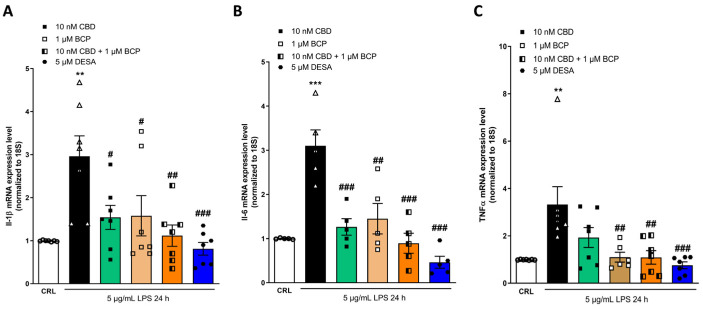
The anti-inflammatory effects of CBD and BCP alone or in combination (1:100) on LPS-stimulated HaCaT cells, evaluated using RT-qPCR. Data shown are the mRNA levels of IL-1β (**A**), Il-6 (**B**), and TNFα (**C**) in HaCaT cells exposed for 24 h to 5 µg/mL of LPS alone or in presence of CBD 10 nM or BCP 1 µM, alone or in combination. The combination induced a significant reduction in the expression levels of IL-1β (**A**), IL-6 (**B**), and TNFα (**C**) compared to LPS. ** *p* < 0.01 and *** *p* < 0.001 vs. CRL, # *p* < 0.05, ## *p* < 0.01, and ### *p* < 0.001 vs. LPS. (ANOVA + Tukey’s w-test). Δ represented LPS alone. ○ represented CRL alone.

**Figure 7:**
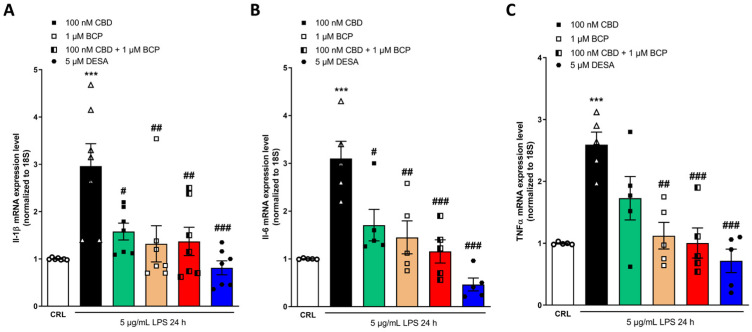
The anti-inflammatory effects of CBD and BCP alone or in combination (at a ratio of 1:10) on LPS-stimulated HaCaT cells, evaluated using RT-qPCR. Data shown are the mRNA levels of IL-1β (**A**), Il-6 (**B**), and TNFα (**C**) in HaCaT cells exposed to 5 µg/mL of LPS for 24 h alone or in the presence of 100 nM CBD or 1 µM BCP, alone or in combination. The combination induced a significant reduction in the expression levels of IL-1β (**A**), IL-6 (**B**), and TNFα (**C**), compared to LPS. *** *p* < 0.001 vs. CRL, # *p* < 0.05, ## *p* < 0.01, ### *p* < 0.001 vs. LPS. (ANOVA + Tukey’s w-test). Δ represented LPS alone. ○ represented CRL alone.

Our data suggested that CBD and BCP act on different targets, so we investigated and compared the efficacy of both compounds, and we explored the advantages of their combination at the doses that were demonstrated to be active in previous studies conducted in inflammatory models. Yokubaitis and colleagues showed the neuroprotective effects of the combination of CBD and BCP in a mouse model of permanent ischemia, and Alonso and co-workers obtained superior results when both compounds were combined, in comparison to the single CBD and BCP administration in an in vitro model of cell inflammation, using LPS-stimulated BV2 cells [36,37]. For these reasons, we studied the combination of 1 µM of BCP and 10 and 100 nM of CBD, at the ratios of 1:10 and 1:100, respectively, in the inflammation model using RT-qPCR, and we evaluated the expression levels of the mRNA of some pro-inflammatory genes such as TNFα, IL-1β, and IL-6. Dexamethasone (DEX) has been used as a positive anti-inflammation control. DEX is a glucocorticoid used to treat different skin diseases [38,39]. These analyses revealed that LPS-stimulated HaCaT cells induced an up-regulation of the pro-inflammatory genes investigated. As reported in Figure 6 and Figure 7, our data showed that both combinations (at both a 1:10 and a 1:100 ratio) of CBD and BCP induced a superior anti-inflammatory effect compared to the administration of the single compound. In particular, the combination of 10 nM CBD and 1 µM BCP, at a ratio of 1:100, induced a significant reduction in the expression levels of IL-1β (Figure 6A) and IL-6 (Figure 6B) compared to LPS. The positive control DEX induced a significant reduction in the expression levels of COX-2 (Figure 6A), Il-1β (Figure 6B), and TNFα (Figure 6C) (all pro-inflammatory genes).

As shown in Figure 7, the combination of 100 nM CBD and 1 µM BCP (at a ratio of 1:100) was able to induce a significant reduction in the expression levels of all the genes that we investigated, as well as TNFα (Figure 7A–C). The positive control DEX induced a significant reduction in the expression levels of COX-2 (Figure 7A), Il-1β (Figure 7B), and TNFα (Figure 7C).

**Figure 8:**
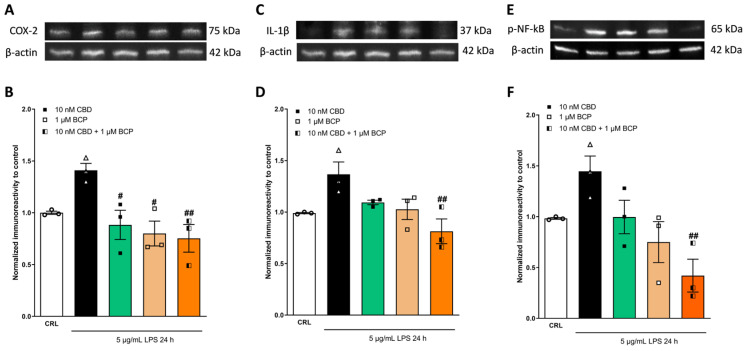
The anti-inflammatory effects of CBD and BCP alone or in combination (at a ratio of 1:100) on LPS-stimulated HaCaT cells, evaluated using WB. Illustrative blots were created using antibodies directed against COX-2 (**A**), IL-1β (**C**), p-NF-kB (**E**), or β-actin. The quantitative analysis of immunoreactive bands (**B**,**D**,**F**) showed that the increased levels of pro-inflammatory proteins induced by LPS were reverted by the combination ratio (1:100). # *p* < 0.05, ## *p* < 0.01 vs. LPS. (ANOVA + Tukey’s w-test). Δ represented LPS alone. ○ represented CRL alone.

**Figure 9:**
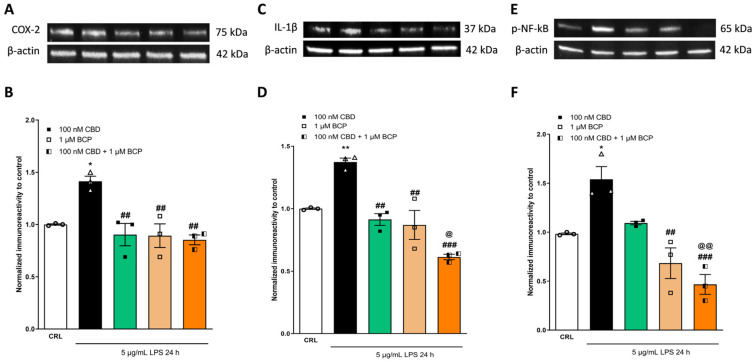
The anti-inflammatory effects of CBD and BCP alone or in combination (at a ratio of 1:10) on LPS-stimulated HaCaT cells, evaluated using WB. Illustrative blots were created using antibodies directed against COX-2 (**A**), IL-1β (**C**), p-NF-kB (**E**), or β-actin. The quantitative analysis of immunoreactive bands (**B**,**D**,**F**) showed that the increased levels of pro-inflammatory proteins induced by LPS were reverted by the combination (at a ratio of 1:100). * *p* < 0.05 and ** *p* < 0.01 vs. CRL, ## *p* < 0.01 and ### *p* < 0.001 vs. LPS, @ *p* < 0.05 and @@ *p* < 0.01 vs. CBD. (ANOVA + Tukey’s w-test). Δ represented LPS alone. ○ represented CRL alone.

To better investigate the additive effects of CBD in association with BCP, which we observed using RT-qPCR, we analyzed the expression levels of inflammatory proteins (COX-2, Il-1β, and p-NF-kB). As shown in Figure 8 and Figure 9, the combination of the drugs induced a major significant reduction in the expression levels of the inflammatory proteins induced by LPS compared to the single drug. The combination ratio (1:10) showed a more significant anti-inflammatory effect (Figure 9).

## 3. Discussion

The human skin is the first organ that protects us from external stimuli that can cause inflammation and different disorders. The keratinocytes are the main cells in the epidermis and are fundamental for skin protection functions and repair during the inflammatory process [17].

Recently, scientific and therapeutic interest in botanical actives, such as *C. sativa* components, for medical and dermatological products has grown. In addition, cannabis compounds are becoming candidates for the treatment of different diseases due to their efficacy and safety profile [40]. The aim of this study was to evaluate the anti-inflammatory properties of CBD and BCP, and of the combination of the two, in the treatment of human skin disease. 

To mimic the inflammatory microenvironment in the human skin in an in vitro model, the human immortalized keratinocyte cell line, HaCaT, was incubated for 24 h with lipopolysaccharides (LPS). The LPS-stimulated HaCaT cells released various pro-inflammatory mediators (COX-2, IL-1β, and p-NF-kB) compared to the control group, as shown in our results in Figure 3 and Figure 4. We used LPS at a concentration of 5 μg/mL for 24 h, because this induces and increases inflammatory markers without toxicity, as previously reported by Tortolani and colleagues [41].

Before investigating the anti-inflammatory effects of CBD and BCP, we performed a cytotoxicity in vitro analysis to confirm their safety profile through a cytotoxicity detection kit (lactate dehydrogenase, LDH), as well as an MTT assay. Any toxic effects were highlighted in the cells treated with CBD and BCP alone or in combination with LPS and the control, as shown in Figure 1. In addition, LPS did not exhibit a toxic effect; this was because they are responsible for the stimulation of the pro-inflammatory process in the HaCaT cells, as shown in the results, without inducing cell death. We used BCP, at concentrations of 1–100 µM, based on the literature; as previously reported by Hye-Sun, BCP at a similar concentration showed anti-inflammatory effects on TNF-α and INF-γ in stimulated HaCaT cells [42]. On the other hand, CBD was utilized at concentrations of 0.001–1 µM, based on the literature and our previous studies, wherein we observed neuroprotective activity of CBD on in vitro models of ischemia and epilepsy [31,32].

Our results showed that both CBD and BCP were able to reverse the LPS pro-inflammatory activity. As shown in Figure 2, CBD demonstrated a dose-dependent anti-inflammatory effect. Indeed, at the concentrations of 0.001, 0.01, and 0.1 µM, CBD induced a significant reduction in the expression levels of COX-2 and Il-1β, and 0.1 µM of CBD induced a significant reduction in p-NF-kB. On the other hand, the 1 µM CBD did not reduce the pro-inflammatory effect of LPS. These results confirmed the pro-inflammatory effect of 1 µM CBD that was formerly demonstrated in other studies, such as the study by Zamansky and colleagues in an in vitro model of HaCaT cells stimulated by TNF-α, or by Juknat and colleagues in a LPS-stimulated BV-2 mouse microglial cell line [43,44]. Also, the lower concentrations of BCP (1–50 μM) demonstrated anti-inflammatory properties, reverting the increase in the expression levels of the inflammatory proteins induced by LPS (Figure 3). Compared to LPS, the 1, 10, and 50 μM concentrations of BCP induced a significant reduction in p-NF-kB levels; in addition, the 1 and 10 µM concentrations induced a significant reduction in COX-2 and Il-1β levels. We analyzed the levels of the pro-inflammatory proteins COX-2, Il-1β, and p-NF-kB because they are released in response to the inflammatory process induced by LPS [17,23,24]. 

To evaluate the mechanisms by which CBD and BCP inhibited pro-inflammatory signaling, using the concentrations that resulted in higher efficacy, we examined LPS-stimulated HaCaT cells co-incubated with CBD and the PPARγ antagonist G3335 and with BCP and the CB2 antagonist receptor AM630. HaCaT cells express the receptors that belong to the endocannabinoid system (ECS), such as CB2 and the PPARγ receptor [45], which can be considered as extensions of the ECS because they can be activated by various lipid molecules, including endo-cannabinoids [46]. CBD and BCP can bind different types of receptors, and the strong involvement of these receptors in the anti-inflammatory process has been demonstrated [47,48]. Additionally, the ECS plays a critical role in human skin; its dysregulation is involved in various disorders, particularly atopic dermatitis, acne, and hyperpigmentation [13]. We decided to co-incubate BCP with AM630, because BCP is a natural selective agonist for the CB2 receptor [25]. We co-incubated CBD with G3335, because previously it had been demonstrated in our laboratory that the beneficial effects of CBD depend on different receptors, including the PPARγ receptor, in rat organotypic hippocampal slices exposed to kainate, which is an in vitro seizure model [32]. In the same study, we performed the experiments with the G3335 and AM630 antagonists; therefore, based on the data of the effect of these drug treatments on the rat organotypic hippocampal slices, and after various attempts on the HaCaT cells, we observed that the G3335 and AM630 antagonists have high efficacy at concentrations of 100 nM. Indeed, as shown in Figure 4 and Figure 5, 100 nM of G3335 and AM630 is able to revert, respectively, the anti-inflammatory effects of CBD and BCP. Our results demonstrated that the anti-inflammatory activity of CBD is modulated by the PPARγ receptor, whereas BCP is modulated by the CB2 receptor, highlighting the pivotal role of these receptors in skin diseases. 

In the literature, there is evidence that the combination of cannabis compounds is more efficient than when they are used alone [49]. For this reason, we decided to complete this study by exploring the anti-inflammatory effects of CBD and BCP in combination. We selected the concentrations of 10 and 100 nM CBD, in combination with 1 µM BCP (at ratios of 1:100 and 1:10). The genes analyzed were IL-1β, IL-6, and TNFα, because they are among the targets of CBD and BCP anti-inflammatory activity [25]. Considering our research, we demonstrated, for the first time, the superior activity of the combination of CBD and BCP in an in vitro model of LPS-stimulated HaCaT cells. Our data showed that (Figure 6 and Figure 7) the combination of CBD and BCP induced a superior anti-inflammatory effect compared to the administration of the single compound. In particular, the combination with the ratio of 1:100 induced a more significant reduction in the expression levels of IL-1β and IL-6 compared to LPS, but the combination with the 1:10 ratio was also able to reduce the levels of the pro-inflammatory gene TNFα. The reason for this may be the action of these natural actives on different targets, namely, the PPARγ and CB2 receptors, which consequently activate two different anti-inflammatory pathways, enhancing their beneficial properties.

## 4. Materials and Methods

### 4.1. Materials

Dulbecco’s Modified Eagle Medium (DMEM), fetal bovine serum (FBS), penicillin and streptomycin, L-glutamine, trypsin-EDTA solution, and phosphate-buffered saline (PBS) were purchased from Merck KGaA (Darmstadt, DA, Germany). Beta-Caryophyllene (BCP) was purchased from Biosfered S.r.l (Torino, TO, Italy) as Endophyllene^®^ P-FL (PNF01), a liquid extract of Piper nigrum. Endophyllene^®^ P-FL was 80% b-caryophyllene in *Oryza sativa* L., and b-caryophyllene concentrations in all experiments were adjusted by 20%. Cannabidiol (CBD) was purchased from ACEF Spa (Fiorenzuola D’Arda, PC, Italy), AM630 was purchased from Tocris Cookson (Bristol, UK), and G3335 was purchased from Cayman (Ann Arbor, MI, USA). 3-(4,5-dimethylthiazol-2-yl)-25-diphenyltetrazolium bromide (MTT) was obtained from Thermo Fisher Scientific (Waltham, MA, USA). The cytotoxicity detection kit (lactate dehydrogenase, LDH) was obtained from Roche Diagnostics (Basel, Switzerland).

### 4.2. Cell Line and Culture Conditions

The human immortalized keratinocyte cell line, HaCaT, comprised spontaneously transformed keratinocytes from histologically normal skin and was purchased from Cell Line Service (CLS, 300493). The cells were cultured in flasks with DMEM, supplemented with 2 mM L-glutamine, 100 µg/mL streptomycin, 100 U/mL penicillin, and 10% FBS (complete medium) at 37 °C in a 5% CO_2_ humidified atmosphere. The medium was changed twice a week. At 80–90% confluence, the cells were detached with 0.025% EDTA 0.5 mM trypsin solution (Sigma, St. Louis, MO, USA) and propagated after appropriate dilution.

### 4.3. Analysis of In Vitro Cytotoxicity

The HaCaT cells were plated and treated with drugs after they had reached the confluence. The medium was used as a positive control, and Triton-X 0.01% was used as a negative control for maximum cell death. Cells were incubated with CBD (0.001–1 μM) or BCP (1–100 μM) for 24 h, and sister cultures were incubated with 5 µg/mL of LPS [41], in the presence or absence of CBD and BCP for 24 h.

### 4.4. 3-(4,5-dimethylthiazol-2-yl)-25-diphenyltetrazolium bromide (MTT) Assay

The viability of HaCaT cells exposed to CBD (0.001–1 µM) or BCP (1–100 µM) for 24 h, and of sister cultures exposed to 5 μg/mL of LPS in the presence or absence of CBD and BCP, was evaluated by MTT assay. The MTT (1 mg/mL) was added to the cells [50] after taking out 100 µL of medium from each well for the LDH assay. The MTT-containing solution was removed, and dimethyl sulfoxide (DMSO) was added to the wells to dissolve the formazan crystal formations. The absorbance of MTT was read at 550 and 690 nm. Cell viability is expressed as a percentage of the cells incubated with the vehicle only, at the corresponding exposure time.

### 4.5. Lactate Dehydrogenase (LDH) Assay

The cytotoxicity in HaCaT cells was measured by the release of LDH into the extracellular fluid, 24 h after drug exposure, as previously described [50]. The LDH level corresponding to complete cell death was determined for each experiment by analyzing sister cultures exposed to Triton-X 0.01%. 

### 4.6. Western Blotting (WB)

At the end of the different treatments, HaCaT cells were washed with cold 0.01 M PBS with a pH of 7.4 dissolved in 1% sodium dodecyl sulfate (SDS). The bicinchoninic acid (BCA) protein assay was used to quantify the total protein levels. Lysates (20 μg/lane of protein) were resolved by electrophoresis on a 4–20% SDS–polyacrylamide gel (Bio-Rad Laboratories, Hercules, CA, USA) and transferred onto nitrocellulose membranes (Bio-Rad Laboratories, Hercules, CA, USA). Blots were blocked for 10 min at room temperature in Every Blot Blocking Buffer (Bio-Rad). After blocking, the blots were incubated overnight at 4 °C with rabbit polyclonal antibodies against IL-1β and COX-2 (all from Abcam, Cambridge CB2 0AX, UK) and with rabbit monoclonal antibodies against Phospho-NF-kB p65 (Ser536) (93H1) (Cell Signaling Technology, Beverly, MA, USA), diluted to a ratio of 1:1000 in TBS-T, containing 5% bovine serum albumin (Sigma-Aldrich, Milan, Italy). As the loading control, the monoclonal anti-β-actin antibody was from Sigma (St. Louis, MO, USA). Immunodetection was performed using secondary antibodies (1:3000 anti-mouse or anti-rabbit IgG from donkey, Amersham Biosciences, Amersham, UK) conjugated to horseradish peroxidase in 20 mM Tris-buffered saline, with a pH of 7.6, and 0.1% Tween 20 (TBS-T) containing 5% non-fat dry milk. Membranes were washed with TBS-T, and then reactive bands were detected using chemiluminescence (ECLplus; Euroclone, Padova, Italy). Quantitative analysis was performed using the QuantityOne—4.6.9 (Basic) analysis software (Bio-Rad, Hercules, CA, USA).

### 4.7. Quantitative Real-Time Polymerase Chain Reaction (RT-qPCR)

At the end of the treatment with 5 µg/mL of LPS, alone or in combination with CBD or BCP, total RNA was isolated from the human immortalized keratinocyte cell line HaCaT using Trizol Reagent (Life Technologies). One microgram of RNA was retrotranscribed using iScript (Bio-Rad, Milan, Italy). RT-PCR was performed as reported previously [50]. The following primers were used: IL-1β human: forward 5′-CAGCTACGAATCTCCGACCACCAC-3′ and reverse 5′-GCCTCGTTATCCCATGTGTCGAAG-3′; IL-6 human: forward 5′-AGCAGCAAAGAGGCACTGGCAG-3′ and reverse 5′-ATCTGCACAGCTCTGGCTTGTTCC-3′; TNF-α human: forward 5′-ACCAAGGTCAACCTCCTCTCTGCC-3′ and reverse 5′-CCAAAGTAGACCTGCCCAGACTCG-3′; 18S human: forward 5′-CGGCTACCACATCCAAGGAA-3′ and reverse 5′-GCTGGAATTACCGCGGCT-3′.

### 4.8. Statistical Analysis

The experiments were repeated at least three times, and the results are expressed as mean ± SEM. The statistical significance of the difference in HaCaT cells’ viability was analyzed using a one-way ANOVA with a post-hoc Dunnett’s test; protein levels and gene levels were analyzed using a one-way ANOVA, followed by the post-hoc Tukey’s w-test for multiple comparisons. All statistical analyses were performed using the GRAPH-PAD PRISM v. 8 for Windows (GraphPad Software, San Diego, CA, USA). A probability value (*p*) of <0.05 was considered significant.

## 5. Conclusions

In the present study, we show the safety profile of the cannabinoid CBD and the terpene BCP in a human epithelial cell line and the anti-inflammatory effects of these compounds alone or in combination. The combination of CBD and BCP allows us to use lower doses of the single components to obtain anti-inflammatory effects, reducing the potential adverse effects associated with the use of higher concentrations. The limitation of this study is the use of the in vitro model; new experiments are required to validate these findings in in vivo scenarios, particularly those related to human skin inflammation, through preclinical and clinical studies. Our data suggest that the topical use of a combination of CBD and BCP should be efficacious for treating skin inflammation and/or other inflammatory disorders.

## Data Availability

Our own data, presented in this study, are available on request from the corresponding author.
